# Cascade: an RNA-seq visualization tool for cancer genomics

**DOI:** 10.1186/s12864-016-2389-8

**Published:** 2016-01-25

**Authors:** Aaron R. Shifman, Radia M. Johnson, Brian T. Wilhelm

**Affiliations:** Laboratory for high throughput genomics, Institute for Research in Immunology and Cancer, University of Montreal, Montreal, QC Canada

**Keywords:** RNA-seq, Visualization, Cancer genomics, Dimensionality reduction

## Abstract

**Background:**

Cancer genomics projects are producing ever-increasing amounts of rich and diverse data from patient samples. The ability to easily visualize this data in an integrated an intuitive way is currently limited by the current software available. As a result, users typically must use several different tools to view the different data types for their cohort, making it difficult to have a simple unified view of their data.

**Results:**

Here we present Cascade, a novel web based tool for the intuitive 3D visualization of RNA-seq data from cancer genomics experiments. The Cascade viewer allows multiple data types (e.g. mutation, gene expression, alternative splicing frequency) to be simultaneously displayed, allowing a simplified view of the data in a way that is tuneable based on user specified parameters. The main webpage of Cascade provides a primary view of user data which is overlaid onto known biological pathways that are either predefined or added by users. A space-saving menu for data selection and parameter adjustment allows users to access an underlying MySQL database and customize the features presented in the main view.

**Conclusions:**

There is currently a pressing need for new software tools to allow researchers to easily explore large cancer genomics datasets and generate hypotheses. Cascade represents a simple yet intuitive interface for data visualization that is both scalable and customizable.

**Electronic supplementary material:**

The online version of this article (doi:10.1186/s12864-016-2389-8) contains supplementary material, which is available to authorized users.

## Background

The growth of next-generation sequencing (NGS) has revolutionized the study of cancer through the analysis of data from whole-exome [1] or genome [2, 3] sequencing, the profiling of epigenetic modifications [4] and transcriptome sequencing (RNA-seq) [5, 6]. RNA-seq is of particular interest, since it is possible to survey gene expression, splicing and mutations in a single experiment. Despite these powerful techniques, a growing list of cancer genomics (CG) studies have demonstrated that defining the precise genetics of the disease remains challenging [7, 8]. Since the molecular mechanisms that can act as “driver events” may differ between patients with the same type of cancer, it is critical for researchers to be able to easily integrate the results of RNA-seq analysis, along with other NGS approaches, to identify functionally equivalent impacts on conserved biological pathways. Software tools that allow researchers to explore their data in an unbiased fashion, to generate potential hypotheses and that are intuitive enough for non-bioinformatians (e.g. clinicians) to apply their specialized knowledge, are therefore critical.

The difficulties associated with the visualization and exploration of multidimensional cancer genomics data have been previously discussed [9], along with efforts to try and address these issues. Ideally, the process of data exploration would also involve representations that utilize an intuitive type of “dimensional reduction” (e.g. with diverse data types being represented by shapes and colours) in order to display a single coherent summary of the information. Moreover, such approaches should also be capable of leveraging the vast amount of verified biological information stored online in resource databases such as the Molecular Signatures Database (MSigDB) [10], Pathway Interaction Database (PID) [11], Reactome [12], and Kyoto Encyclopedia of Genes and Genomes (KEGG) databases [13].

Here we present Cascade, a new data visualization tool to display and explore NGS datasets in a rapid and intuitive way by allowing multiple data attributes to be shown simultaneously. Cascade allows the analysis of RNA-seq data, or whole-exome or genome sequencing, to be easily mapped onto known or user defined biological pathways. The program uses a variety of tunable parameters to highlight specific attributes of genes/features that are of interest to the researcher. Cascade can also easily integrate other data sources and, through user configurable gene lists and networks, highlight relevant features of the data. By allowing researchers and clinicians with specialized information to browse and explore their data in the context of existing biological knowledge, Cascade fills a gap which currently exists between tools used initially to process the data for highly specific tasks (read-mapping, variant detection) and tools that allow reanalysis of published data.

## Implementation

**Fig. 1:**
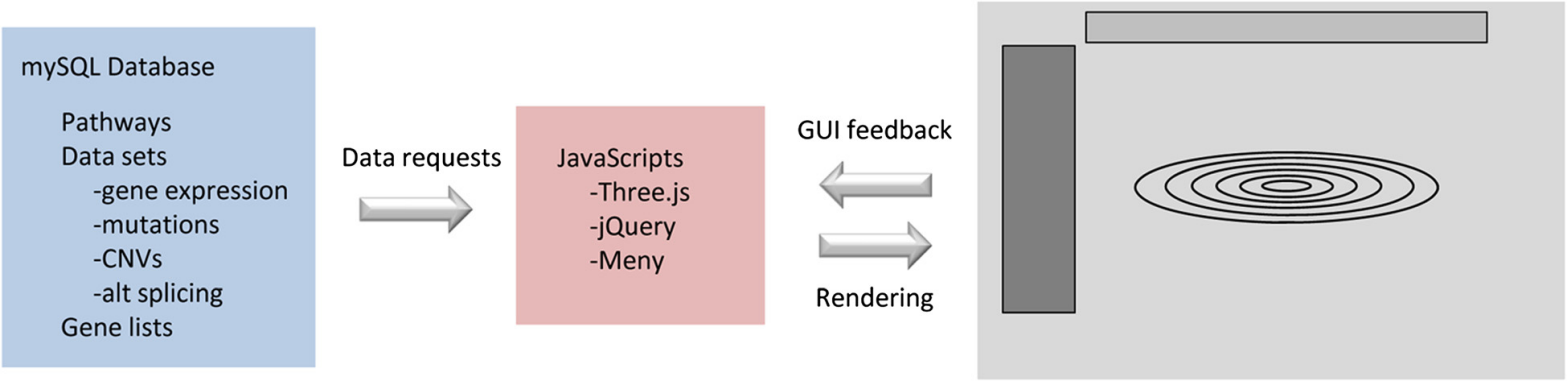
Overview of Cascade organization. A cartoon view of Cascade is shown with the three principal components coloured. The relational database (*blue*) holds all user defined data (e.g. from RNA-seq experiments) along with pre-defined data for biological pathways, networks, disease associated genes, etc. A collection of Java/PHP scripts (*pink*) act as bridge between the database and the main webpage (*grey*) used for user interaction and data visualization

Cascade is a web-based user interface that allows researchers to interactively explore their RNA-seq data while allowing a wide variety of data types to be displayed. Cascade consists of the main web page, an underlying relational database (MySQL) containing all of the information from RNA-Seq experiments (along with information defined in biological pathways, gene lists, etc.) and a collection of PHP scripts allowing the user to submit requests to the database to be displayed in the browser (Fig. 1).

**Fig. 2:**
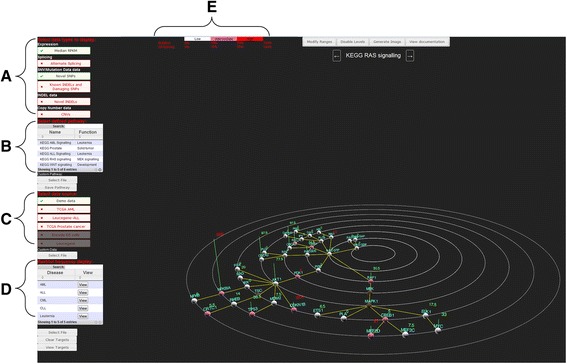
Screenshot of data rendering on main webpage. Cascade uses a space-saving menu on the left-hand side of the screen to store functions to: (**a**) select features of the RNA-seq data to be displayed, (**b**) select biological pathways to overlay data onto, (**c**) select datasets to use for visualization of features selected (using **a**) and (**d**) restrict the colouring thresholds for features based on custom or predefined disease gene lists. The “modify ranges” button (*top centre*) allows users to alter the cohort frequency thresholds (**a**) required for node colour changes (*mutations*) or ring appearance (*splicing*). Additional buttons (*top*) toggle display of guide rings, generate (*.jpg*) screen images or open tool documentation

The main Cascade webpage uses the scripts from the Java library Three.JS [14] to generate interactive 3D forms without a requirement for specific browser plugins. The main page is sub-divided into a plotting canvas where the genes (nodes) and interactions (edges) are rendered, a space-saving, collapsible, menu on the left-hand side of the application [15] and a fixed information ribbon at the top of the page (Fig. 2). The rendering canvas depicts genes as spheres on a single plane linked by edges/lines (interactions) with concentric rings as an arbitrary guide for pathway depth (which can be toggled on/off). Gene names are shown above each node and gene expression values are represented as green vertical lines. The functionality of Three.JS is completely generic and therefore although the planar view is a default representation (since many established pathways have a sequential order of interactions), the Cascade source code could be modified to also allow the representation of typical “hair-ball” network diagrams. Cascade graphics are also entirely customizable by end-users and some modifications (e.g. colour selection, font size) can be made directly through a simple file of parameters.

In order to optimize the use of screen space, a collapsible side-menu (Fig. 2) to view the data selection options is displayed on the left side of the page. The menu allows users to alter 4 aspects of the rendered view: A) the data types to include in the representation, B) the biological pathway to overlay data onto, C) the source of the RNA-seq data, and D) the gene list selection to restrict frequency colouring for the data types selected. The choice of data types to represent is dependent only on what is available in the database or uploaded by the user, allowing individuals to tailor the view to their needs. Although Cascade could easily be altered to display any type of data (gene lengths, %GC, etc.), has been designed for cancer genomics data. Therefore the default data types available to be shown (if available) are copy number variations (CNVs), single nucleotide variants (SNVs; either novel or known damaging variants), gene expression (either mean/median levels of gene transcription), alternate splicing, and insertion/deletions (indels). For instance, activating the “alternative splicing” option will add coloured rings around nodes based on whether or not that gene has been flagged by the user as exhibiting alternative splicing. User supplied values are required for this which could represent a binary observation of alternative splicing or a skewed ratio of isoforms since individual isoforms cannot be represented. Likewise, the presence of known/predicted deleterious SNVs must be assigned by the user when the data is loaded into the database in order for it to be displayed properly and for cohort frequencies to be calculated properly. The second input required form the user is to define the biological pathway to overlay the RNA-seq data onto. A number of predefined pathways from KEGG have been entered and Cascade will also accept user-generated files of pathways. A text search box in the upper right allows restriction of shown pathways based on gene name or function title. In order to allow rapid browsing of RNA-seq data in multiple pathways Cascade also allows for a “quick-scroll”. This can be accomplished by left clicking on the left/right arrows next to the current pathway name or by a right mouse button click-drag anywhere on the screen. A click-drag change in horizontal position of more than 200 pixels (similar to a “swipe” gesture on a smart phone) will trigger the transition from the current pathway to the next (either before or after, depending on the click-drag direction). The third user option on the menu is the RNA-seq data source which can be selected simply by clicking on the appropriate box in the section, with the current data source shown highlighted in green. Lastly, the fourth option, which is to restrict the colour frequency displays to specific genes, can be activated by clicking on a specific gene list. Because the inclusion of novel SNVs in a large cohort can have the effect of “swamping” the view with coloured nodes, the gene list restriction allows users to restrict the colouring to only a specific list of genes (independent of which pathway is being examined). Several default lists of genes implicated with specific diseases are provided, but users can enter their own gene list using the “Select File” option below the lists. Gene lists can also be selected based on the presence of specific genes names through the textbox search tool similar to the pathway list.

Cascade is designed to calculate and summarize feature information from a cohort of samples in the rendered network view through a node/shape colouring scheme. When an experimental data source is selected from the side menu, the data is loaded from the database and client side calculations are performed to generate statistics, including gene expression distribution (mean, median), and mutation/alternative splicing frequencies. The resulting statistics are then used to alter the colour/rendering of the nodes within the current network based on the user-defined parameter values. For frequency based values, the data is partitioned into three bins using user defined limits and a gradient color scheme. This allows users to differentiate between mutations which are rare/absent (“low” in white), infrequent (“intermediate” in pink) and common (“high” in red) and highlight changes which are of most interest. The numerical limits of the three bins can be altered by selecting the “modify ranges” button at the top of the page in the fixed information ribbon. Because these cohort-based calculations are performed on the client side, large cohorts/networks (>200 samples) can result in somewhat slower rendering times and therefore should be performed on relatively modern workstation (e.g. >8GB RAM, and a discrete graphics card) running any up-to-date browser which supports WebGL.

**Fig. 3:**
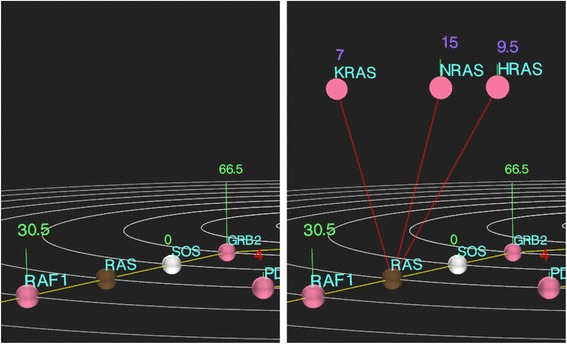
Visualization of gene families. In order to reduce the complexity of the gene view in Cascade, families of closely related genes can be represented by single nodes in the main rendering view and expanded by left clicking on the “parent” node. The child nodes are then shown, with appropriate colouring and information according to the user thresholds, on a second elevated plane. Left-clicking on these child nodes reveals the same tabbed window for individual genes as on the main plane

In order to allow the user to control the complexity of the pathway view, Cascade also has the capability of rendering gene families, such as the Janus kinase family (JAK1, JAK2, JAK3, and TYK2) for instance, as a single (brown) node. These family nodes do not show data values by default, but they can be expanded by left-clicking on the node which then plots the child nodes within the family above the plane of the network with their respective colouring and information (Fig. 3). Once a gene of interest has been identified, left clicking the node will open a new vertically tabbed window. The default “About” tab contains gene information that is dynamically retrieved from RefSeq entries provided the proper HUGO gene symbol is used in the pathway. Additional tabs allow more detailed views of expression patterns, mutation/SNP presence, alternative splicing and copy number variations (CNVs) on a per sample basis.

### PHP Scripts

The server-side components of Cascade are run on an Apache web server supporting PHP, while the back-end scripts act as an interface between the client and the server. The server-side scripts used by Cascade belong to two categories, either server retrieval or server storage. The scripts for data retrieval return one of the pre-existing pathways from the database, including the genes within the network, their connections, and the keyword descriptions of the pathway. Additional retrieval scripts can return detailed sample information based on the gene name, in addition to lists of genes associated with specific diseases. In order to allow greater flexibility for users, Cascade is currently designed to save novel, user-defined, pathways (added via a file upload) to the server directly from the main webpage. This option transfers the pathway information (genes, connections, pathway category) to the server, making it available for use by other users. Other user-defined information (additional genes, expression data, etc.) can be added to the database but this requires direct access (e.g. using MySQL Workbench, MySQL plugin for excel) rather than through using the webpage.

### JavaScripts

Cascade is built using the Three.JS framework (3JS), a Javascript library designed to provide fast, efficient three dimensional web rendering functionality for the user interface. In order to render a 3D scene, 3JS requires three elements: a camera, a model, and a light source. Because 3JS allows generic and abstract visualization, the model used by Cascade is an interconnected gene network and is defined through a series of 3D vectors and shapes. Each shape in the view has an associated material and attributes (referred to as “optical properties”) that define how it is rendered. These properties can be used by Cascade to define elements of interest (e.g. through colour changes) based on user-defined criteria. The “camera” in Cascade represents a mathematical operation defining the perspective view of the model. The view in Cascade is defined by several distance parameters that allow the perspective to be dynamically recalculated as the user pans or zooms within the view. Within the Cascade view, light sources have been added to create an even level of illumination regardless of the 3D perspective, making it easier to see the colours applied to shapes.

### Database

The Cascade demonstration website uses a mySQL relational database with an InnoDB transaction model, allowing foreign key constraints on the database and by extension, each pathway. Tables with pathway information are linked by a series of cascading index keys which allows for more efficient information retrieval. Cascade queries are sent to the server to retrieve a pathway through the pathway table, allowing the subsequent retrieval of the complete network sequence for rendering (Additional file 1: Figure S1).

All user-supplied biological data is also stored in the database with each data type (mutations, expression, CNVs and alternate splicing data) in separate tables and linked by unique sample identifiers. When Cascade performs a server call for data, the server receives a list of genes and samples based on the pathway and data source selected, and returns the corresponding data, if available, for calculation and rendering. Cascade does not require all data types to be available in order to visualize the existing data. On the other hand Cascade does not evaluate the nature of the data that is assigned to each pre-defined category. In principle, this allows extensive user driven customization through very minor alterations in the HTML code, for instance replacing “alternative splicing” with “promoter methylation”. Aside from a mandatory sample identifier (e.g. patient number), the format of each table is simply defined by the data type stored: binary values (e.g. 1/0, true/false) for mutations and alternate splicing, or a numeric value (e.g. 2, 3.8) for CNVs and expression.

**Fig. 4:**
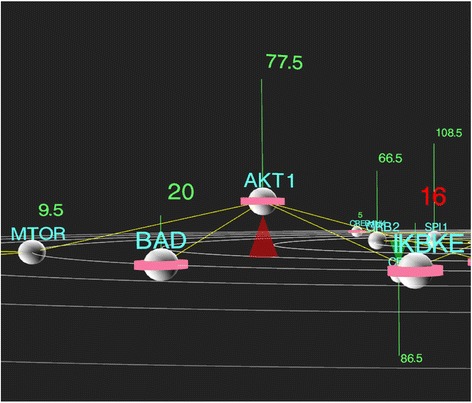
Screenshot of CNV and splicing representation. Copy number variations (CNVs) are represented in the Cascade display as gene nodes raised or lowered from the plane of the pathway by a height which is proportional to the value of the average CNV value within the samples set. Copy number gains are shown as nodes onto top of red cones, which copy losses as shown as genes on green cones. Alternative splicing frequency within a sample cohort is represented by a ring around the node which is coloured according to the frequency thresholds defined by the user

In order to facilitate the identification of subgroups within data, expression values are evaluated for deviations from the sample means (through user adjustable values in the source code), such that genes which contain at least a certain percentage of outlier samples will be indicated by a red expression value at the top of the vertical line (typically RPKM values (**R**eads **P**er **K**ilobase of transcript per **M**illion mapped), but any numeric expression values could be used with minor adjustments). Similarly, user-supplied alternate splicing values for genes are represented as a ring around the gene node (Fig. 4), with coloring reflecting the frequency of splicing in the same way as mutation frequency. Lastly, copy number variations (CNVs) are represented as gene nodes that are positioned above or below the plane, depending on whether there are copies gained (above) or lost (below), on average, in the data set (Fig. 4). CNVs can either be displayed as Boolean values (constant +/−), or in relative values where the node deviation represents the mean CNV value, through multiple clicks on the CNV button.

## Results

The current web-accessible version of Cascade contains published cancer genomics data from either ALL (Leucegene [16]), AML or prostate (TCGA [7, 8]) RNA-seq studies as well as demonstration data used to highlight functionality of the software. While the data currently accessible from Cascade provides the basis for visualizing different aspects of RNA-seq experiments, the nature of how the data is treated is quite flexible. For instance, in the demonstration data, the CNV option currently presents an average of the store values for each gene. Because the values in actual sample data may represent a more homogenous group with a few outliers (amplifications), this display mechanism may not be ideal, since the average CNV within the cohort might produce only a small elevation which could mask relevant differences. A simple alternative is to simply use a frequency threshold (as for mutations) where a cone of non-meaningful height is added as a flag to highlight genes that pass this threshold. Users can then click on specific gene nodes to examine the CNV data under the specific tab for specific targets of interest. Therefore, while Cascade is current a functional stand-alone tool, various aspects of its functionality can tailor the output to suit the needs of individual research labs.

## Use case for T-ALL

**Fig. 5:**
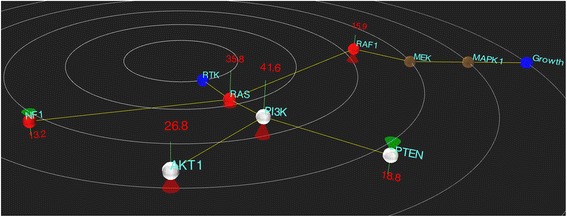
An example use-case of Cascade. A representation of the biological pathway for MAP2K4 published by Zhengyan et al. is shown with CNV and mutation data adapted from the analysis of ovarian cancers. The increased frequency of CNV gains in oncogenes (e.g. AKT, PI3K, RAF) and loss in tumor suppressors (e.g. NF1, PTEN) are evident while mutations in oncogenes and tumor suppressors (*red nodes*) are also displayed

In order to assess the usefulness of Cascade for cancer genomics data exploration we generated a number of biological pathways based on KEGG information and also entered a number of published RNA-seq datasets into the Cascade database. As an initial confirmation of function, we examined a previously published analysis of ovarian cancer data by Zhengyan et al. [17]. In this paper the somatic mutations present in 441 tumors from 4 tissues were examined and MAP2K4 (involved in JNK signalling) was characterized in more detail. Using a pseudo-cohort of patient data with equivalent frequencies for the CNV changes and mutations, along with a generic RTK pathway, the view in Cascade clearly showed copy number increases of oncogenes and losses of tumor-suppressors, along with a higher frequency of mutations in a number of the genes in the pathway (Fig. 5). This demonstration provides a simple example of how Cascade can present an integrated view of various data types.

**Fig. 6:**
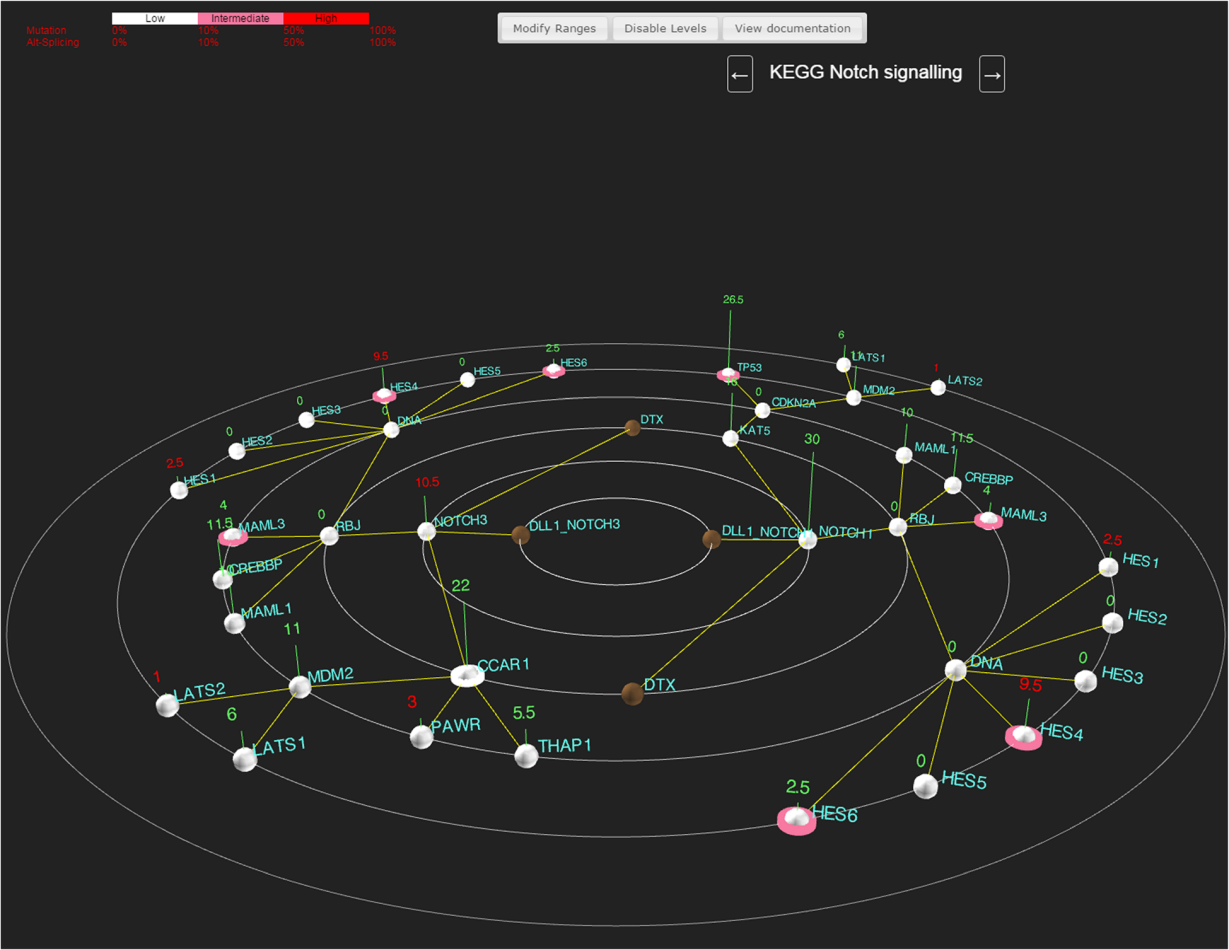
Notch signalling in Cascade. A NOTCH signalling pathway was designed and loaded into Cascade and previously published T-ALL data \was overlaid on to the pathway. Thresholds for mutations and splicing were left at default values and visualizing of splicing and CNVs was activated

We next re-examined human T-ALL data published recently [16] to look for novel insights using Cascade. In the original publication, the leukemogenic role of CNVs and mutations in CDKN2A and NOTCH1 were respectively examined. To complement the expression and mutation data for these samples, we also added data for alternative splicing (generated using MISO [18]) to the dataset in Cascade. We then generated pathways based on KEGG interactions along with recent publications for NOTCH signalling [19, 20] (Fig. 6) since mutations in the *NOTCH1* gene essentially divide the dataset in two.

**Fig. 7:**
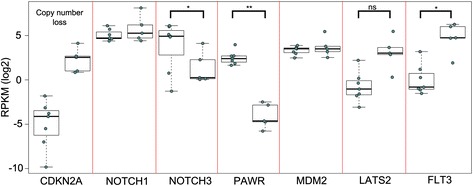
Gene expression differences based on CDKN2A loss. Boxplots of the expression level (log RPKM) of 7 genes implicated in NOTCH signalling are shown, with individual patient RPKM values being shown as dots. For the expression of all genes, patients were grouped based on their copy number loss of CDKN2A (first column) which ablates gene expression. In cases where differences were observed, the significance (based on a Welch two sample *t*-test) is shown (ns = not significant, * = *p* < 0.05, ** = *p* < 0.01)

**Fig. 8:**
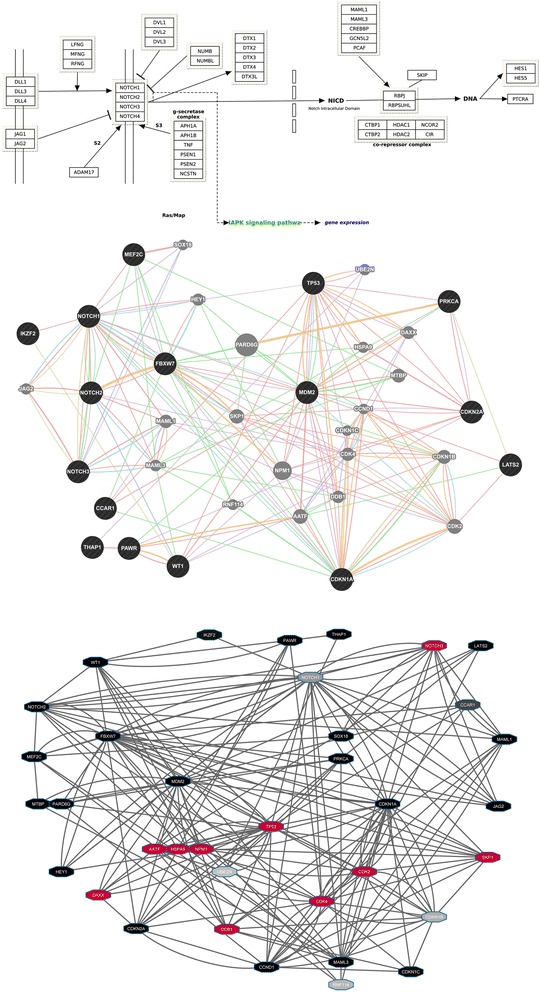
Alternative NOTCH pathway representations. NOTCH signalling pathways are shown that were either pre-defined through manual curation of literature (**a** – from KEGG) or generated dynamically through known genetic and physical interactions along with other molecular properties (**b** – from GeneMania). The interaction network defined by GeneMania was saved an imported into Cytoscape (**c** - ver.3.2.1) and T-ALL patient RPKM values were used to colour nodes based on *high*/*medium*/*low* (*red*/*grey*/*black*) expression level

In examining the view of the Notch pathways, we observed that the expression of *NOTCH3* was highlighted as containing outliers (along with *PAWR*). While *PAWR* and *THAP1* have been implicated in the alternative splicing of *CCAR1* [20] (which Cascade shows occurs a low frequency with default settings), the correlation of *NOTCH3* expression levels with other anomalies has not been specifically investigated in cohorts of T-ALL patients. Using Cascade’s “gene expression” tabs for the *NOTCH3* and *PAWR* nodes reveals a highly dichotomous expression pattern for the same sets of patients. Interestingly, expanding the CNV tab for the previously published *CDKN2A* copy number loss and sorting on the relative copy number shows that the patient samples with a copy loss of *CDKN2A* also have higher expression of *NOTCH3* and *PAWR*. Although other pathway members (e.g. *NOTCH1, MDM2*) do not change in expression as a result of the *CDKN2A* CNV, other genes highlighted with outliers (e.g. *LATS2, FLT3*) show the reverse pattern (also shown in Fig. 7). These consistent differences suggest that there is a specific biological response in T-ALL samples with *NOTCH1* mutations and loss of *CDKN2A* that potentially involves enhanced *NOTCH3* expression. This has previously been suggested to be important for T-ALL, but not in combination with genetic anomalies in *NOTCH1* and *CDKN2A* [19]. The higher expression levels of other signalling proteins (e.g. FLT3) seen in *NOTCH1* wt patients suggests that in these patients, alternative pathways for activation may be critical. It is also of interest to note that *MAML3* shows alternative splicing in 2 samples (both of which lack the *CDKN2A* CNV) since the altering splicing of MAML factors has been found to impact NOTCH signalling in flies [21]. Taken together, the observations in Cascade using an integrated view of gene expression, splicing, CNVs and mutations highlight several potential novel connections related to T-ALL signalling through the NOTCH pathway. While other views of this signalling pathway or network can be generated (Fig. 8), they lack the ability to integrate and represent all of the data as Cascade does, making it far less likely that observations above would have been clearly evident.

## Discussion

Cancer genomics RNA-seq experiments, along with whole genome/exome sequencing, have rapidly evolved into standard methodologies for the characterisation of tumors in both clinical and research contexts. This has resulted in the rapid proliferation of bioinformatics tools to optimize read mapping (reviewed in [22]), mutation/variant detection (reviewed in [23]) and identification of chromosomal fusions [24]. Despite this, the interpretation of cancer genomics data for large cohorts still represents an unresolved challenge for researchers looking for common genetic mechanisms in the disease. Furthermore, while a wide range of more targeted software has developed for earlier analytical stages, there has been significantly less emphasis on tools for integrated visualization of the resulting data.

Currently, there are a number of tools available to visualize specific aspects of the data (reviewed in more detail elsewhere [9, 25]), which can be broadly grouped into three classes: track based viewers, network based viewers, or data analysis/retrieval portals. Track based tools such as IGV [26], UCSC genome browser [27], GBrowse [28], Ensembl [29], and Artemis [30] allow for the visualization of read mapping coverage and mutations, and continuous value characteristics (e.g. enrichment data for ChIP-seq, DNA methylation, chromatin accessibility, etc.). Track-based viewers are generally most useful for the visualization of individual genes or loci (e.g. to validate mutations), in order to simultaneously view several data types (e.g. gene expression data with ChIP-Seq data). While useful for specific applications, the ability to view large numbers of samples or data types (while maintaining all samples in a single view) is practically limited by the screen space available for representation of tracks. Network based data visualization tools such as Cytoscape [31] or Genemania [32] alternatively allow users to generate a generic two-dimensional (or three dimensional with BioLayout Express 3D [33]) representation of interactions (e.g. genetic or protein) shown as nodes connected by edges. These tools offer more flexibility with respect to the way in which the data is organized and represented and can typically overlay gene expression data. In spite of this, because the primary use of these programs was not to simultaneously represent the full range of RNA-seq data types, their functionality can impose limits on how the data can be analyzed. Lastly, other dedicated CG analysis tools such as cBioportal [34] and the UCSC Cancer Genome [35] browser offer data visualization services along with a number of integrated analysis tools. This approach permits researchers to examine already published patient cohorts although both tools are largely “gene-centric” and require the user to enter specific gene names in order to examine these specifically. Other cancer genomics data portals such as the TCGA cancer genomics hub (https://cghub.ucsc.edu/) or visualization tools such as Mapman (http://mapman.gabipd.org/web/guest/home), or Gittools (http://www.gitools.org/) also address some specific concerns but do not represent generic solutions for visualization. Overall, while all of the software described above can be useful for cancer genomics, tools that allow data exploration and hypothesis generation from unpublished data are still lacking.

We have developed Cascade as novel tool to address this need and aid researchers with the exploration of their RNA-seq data. Cascade is designed to allow a sort of “dimensionality reduction” of a data set (e.g. one view to represent 4–5 data types) while still allowing users to easily access the underlying data for each sample. This ability is critical, since presenting an average gene expression value for a particular gene could, for instance, hide a bimodal distribution pattern of expression values.

Although the importance and utility of data visualization for large datasets is clear, there has historically been a very strong bias against using three dimensional approaches to do this [36]. Two of the principle arguments against the use of 3D approach (for data that is not inherently structural, such as protein crystal structures) are the occlusion of objects in the field of view and visual misrepresentation of data due to differences caused by a 3D perspective projected in 2D. Although such problems can be highly problematic in static 3D views, in Cascade the view can be easily rotated and zoomed and therefore this problem is largely obviated. Indeed, a rotatable or dynamic view has been suggested to address exactly this problem [36]. The problem of apparent size differences created by the view perspective is however inherent in any 3D viewer. Therefore, Cascade displays in addition to the vertical bars numerical RPKM values to remove potential perspective based problems. In addition, no other object in Cascade uses size -encoding to represent data. One last concern with 3D visualizations relates to colours, shading and lighting that can be influenced by “shadows” cast by objects in the view. Because the node sizes used by Cascade are small and relatively distant and the view has uniform and multidirectional lighting, the colour-encoded data is clearly visible. In summary, Cascade was designed specifically to avoid many of the main concerns of 3D data visualization tools.

Through our use case for T-ALL, we show that Cascade can help to address this need and while the potential hypotheses generated (NOTCH3 activation being linked to *NOTCH1 /CDKN2A* loss, alternative activation of FLT3 along with a potential role for alternative splicing of *MAML* members in *NOTCH1 wt* patients) would require experimental validation, the goal of Cascade is simply to allow users to rapidly generate novel hypotheses based on their data. Given the incredible volumes of complex cancer genomics data being generated, it is increasing critical that researchers have access to such simple and intuitive tools to explore their data in an unbiased fashion. The use case presented above also reflects the importance of being able to leverage existing knowledge (e.g. biological pathways, interactions, etc.) to be able to uncover novel biological relationships. Although no single tool will reveal the biology of cancer, Cascade allows users to tailor the view of their data, making it simpler to focus on specific combinations of biological phenomena (expression and splicing, CNV and expression, mutations and splicing, etc.) which may be relevant.

While the primary goal for the development of Cascade was to provide an intuitive interface for visualizing data, it also allows end-users considerable flexibility to customize the view to suit their data as discussed above. Therefore while a number of common data types are designed into the Cascade viewer, the data type represented (including the HTML labels shown in Cascade) can easily be altered, providing a more generic and customizable tool. Since the Three.JS library can present any type of shape, material, lighting, etc. the basic framework of Cascade could easily be extended to include additional information regarding regulatory relationships (e.g. along edges) or additional forms of data (e.g. DNA methylation). Additionally, the interface can easily be modified to display data from other organisms (e.g. bacteria, plants, etc.) or for other experiment types (e.g. metabolomics) with appropriate user-supplied annotation. By hosting the Cascade code within a central repository, the expectation is that this will facilitate the incorporation of new functionality into the standard release versions.

## Conclusions

As an ever-increasing amount of data from cancer genomics projects becomes available, the development of tools to allow the exploration of this data is becoming a critical priority. Cascade is a novel tool for the visualization of RNA-seq data from cancer genomics projects, which has the advantages of providing researchers with an intuitive interface for exploring their data in the context of known biological pathways. In addition, Cascade is designed to be easily customized with respect to both the data and the display, ensuring that atypical data types can be represented with the Cascade framework. The planned continued development of Cascade will add functionality for analysis and comparison of datasets, allowing researchers and clinicians to easily transition from data exploration to hypothesis generation.

## Availability and requirements

Project name: Cascade

Project home page: http://bioinfo.iric.ca/~shifmana/Cascade/

Source code: https: https://github.com/aaronshifman/Cascade_RNAseq_viewer

Operating system(s): Web based, Platform independent.

Programming language: HTML5: HTML + CSS + JavaScript

Other requirements: modern browser (2012+), 2Gb RAM

License: GPL2

Any restrictions to use by non-academics: None

### Ethics

No ethics approval was required for the use of the clinical data used in this study.

## Additional file

Additional file 1: Figure S1. Cascade database schema. The database schema for Cascade is shown with boxes differentiating the three principle data types. The tables in part A represent the user supplied RNA-seq results including gene expression, mutation status, CNVs and alternative splicing. The tables in section B represent annotation files for genes and transcripts in addition to insertions and deletions (Indels) and single nucleotide polymorphisms (SNPs) and their consequences. The last section (C) contains tables with information for predefined or custom biological pathways, the genes involved in the pathways, pathway annotation as well as disease associated gene lists. (PDF 1149 kb)

## Abbreviations

CG: Cancer genomics
CNV: Copy number variation
NGS: Next generation DNA sequencing
GB: Gigabyte
indel: insertion or deletion
RAM: Random access memory
RPKM: Reads per kilobase per million reads sequenced
T-ALL: T-cell acute lymphoblastic leukemia
wt: Wild-type
