# Supplementary figures and images for: Cascade: an RNA-seq visualization tool for cancer genomics
Source: BMC Genomics. 2016 Jan 25;17:75. doi: 10.1186/s12864-016-2389-8 (PMC4727405; doi:10.1186/s12864-016-2389-8)

# Cascade mySQL DB

A

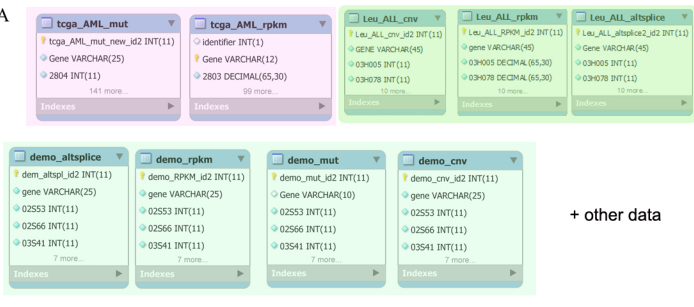

B

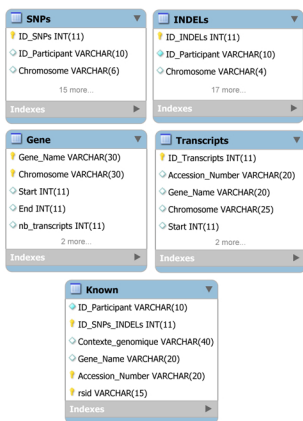

C

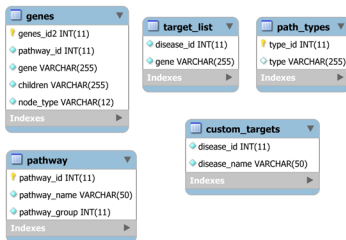

Supplement: Additional file 1: Figure S1. — Cascade database schema. The database schema for Cascade is shown with boxes differentiating the three principle data types. The tables in part A represent the user supplied RNA-seq results including gene expression, mutation status, CNVs and alternative splicing. The tables in section B represent annotation files for genes and transcripts in addition to insertions and deletions (Indels) and single nucleotide polymorphisms (SNPs) and their consequences. The last section (C) contains tables with information for predefined or custom biological pathways, the genes involved in the pathways, pathway annotation as well as disease associated gene lists. (PDF 1149 kb) [file 12864_2016_2389_MOESM1_ESM.pdf]
